# Effect of Laparoscopic Roux-en-Y Gastric Bypass Surgery on Obstructive Sleep Apnea in a Chinese Population with Obesity and T2DM

**DOI:** 10.1007/s11695-014-1510-9

**Published:** 2014-11-14

**Authors:** Jianyin Zou, Pin Zhang, Haoyong Yu, Jianzhong Di, Xiaodong Han, Shankai Yin, Hongliang Yi

**Affiliations:** 1 Department of Otolaryngology, Shanghai Jiao Tong University Affiliated Sixth People’s Hospital, Otolaryngology Institute of Shanghai Jiao Tong University, 600 Yishan Road, Shanghai, 200233 China; 2 Department of General Surgery, Shanghai Jiao Tong University Affiliated Sixth People’s Hospital, Shanghai, 200233 China; 3 Department of Endocrinology and Metabolism, Shanghai Jiao Tong University Affiliated Sixth People’s Hospital, Shanghai Clinical Center for Diabetes, Shanghai, 200233 China; 4 Shanghai Diabetes Institute, Shanghai Key Laboratory of Diabetes Mellitus, Shanghai Key Clinical Center for Metabolic Disease, Shanghai, 200233 China

**Keywords:** Obstructive sleep apnea, Bariatric surgery, Diabetes mellitus, Obesity, Prediction model

## Abstract

**Background:**

Bariatric surgery has been reported to be an effective treatment for obstructive sleep apnea (OSA). However, this evidence was not enough for different populations. Thus, we conducted a follow-up study to evaluate the effect of bariatric surgery on OSA in a Chinese population with obesity and type 2 diabetes mellitus (T2DM).

**Methods:**

From May 2011 to March 2014, 72 consecutive subjects with obesity and T2DM were recruited for this study. Before and at least 6 months after the laparoscopic Roux-en-Y gastric bypass (LYGB) surgery, all subjects were asked to undergo a polysomnography test. During the sleep center visit, anthropometric characteristic data, blood samples, and sleep questionnaires were collected.

**Results:**

In total, 44 Chinese participants with OSA were included in the study. Compared with baseline data, the postoperative anthropometric characteristics, blood measurements, and sleep recording data, such as weight, apnea hypopnea index (AHI), and insulin resistance index, differed significantly (*p* < 0.001). The change in AHI was correlated significantly with preoperative weight (*r* = 0.298, *p* < 0.05), preoperative AHI (*r* = 0.729, *p* < 0.001), preoperative waist circumference (*r* = 0.307, *p* < 0.05), and preoperative insulin resistance (IR) index (*r* = −0.301, *p* < 0.05). Postoperative AHI was correlated significantly with age (*r* = 0.039, *p* = 0.039) and preoperative AHI (*r* = 0.445, *p* = 0.002), and the following prediction model was generated: log_10_ (postoperative AHI) = 0.626 × log_10_ (preoperative AHI) +0.010 × age −0.581.

**Conclusions:**

Our findings indicate that LYGB could be an effective therapeutic intervention in the management of OSA for patients with both obesity and T2DM, and the preoperative AHI and age might be important factors that influence the effort of LYGB.

**Electronic supplementary material:**

The online version of this article (doi:10.1007/s11695-014-1510-9) contains supplementary material, which is available to authorized users.

## Introduction

Obstructive sleep apnea (OSA) is one of the most common sleep disturbances; it affects 3.2–20 % of the adult population [1, 2] and has a negative impact on public health, increasing both morbidity and mortality [3]. Although continuous positive airway pressure (CPAP), oral appliances, and surgical modifications of the airway are considered parts of the routine management of patients with OSA, there remain many problems with these treatments in practice. Nasal CPAP is considered the gold standard therapy for OSA [4]. However, reported CPAP compliance rates vary from 34 to 85 % [5, 6], which might limit the therapeutic value for OSA patients over the long term. Oral appliances are generally viewed as being less efficacious than treatment with CPAP [7, 8]. Moreover, 6–86 % of patients who used oral appliances have been reported to experience adverse effects, including dryness of the tongue and throat, pain in the teeth and jaw, and insomnia [7, 9]. Various surgical techniques of the upper airway have been used to treat OSA, although the use of surgery for this condition remains somewhat controversial and is associated with limited and unpredictable efficacy that may diminish over the long term [10–12]. That is, although several treatments exist, they are often poorly tolerated or only partially alleviate the abnormalities. Thus, there is a need to improve patient adherence to existing treatments and to develop new treatments.

Bariatric surgery, which is currently the most effective treatment for morbid obesity, has been reported as a novel therapeutic treatment option for OSA [13]. The effect of bariatric surgery on OSA has been reported to be encouraging [14, 15]. However, these published series have not included diverse populations, especially in developing countries. Moreover, whether bariatric surgery is applicable to patients with OSA and other complications, such as obesity and type 2 diabetes mellitus (T2DM), should be determined.

Given the significant morbidity and mortality associated with OSA and the potential therapeutic value of bariatric surgery, we conducted a follow-up study of the effect of laparoscopic Roux-en-Y gastric bypass (LYGB) surgery in a Chinese obesity population with both OSA and T2DM.

## Methods

### Participants and Measurements

From May 2011 to March 2014, 72 consecutive subjects with obesity and T2DM, who chose to be treated with LYGB surgery in our hospital, were recruited for this study. The diagnostic criterion for obesity [16] was a body mass index (BMI) of 25 kg/m^2^. The diagnosis of T2DM was based on the 1999 World Health Organization criteria [17]. All participants were aged 20–70 years. Patients with psychiatric disturbances and those undergoing systemic steroid treatment or hormone replacement therapy were excluded.

All participants provided written informed consent. This study was approved by the Ethics Committee of the Shanghai Jiao Tong University Affiliated Sixth People’s Hospital and complied with the Declaration of Helsinki.

Before and at least 6 months after the LYGB surgery, all subjects were asked to undergo an overnight PSG test in the sleep center at our hospital. During the sleep center visit, all participants were asked to report whether the witnessed apnea exist and completed the Epworth Sleepiness Scale (ESS) questionnaire before the overnight PSG test. Fasting blood samples were taken the next morning for measurement of glucose and insulin concentrations. Body habitus, including weight, height, neck circumference (NC), waist circumference (WC), and hip circumference (HC), was measured using standard anthropometric methods.

### Polysomnography and Definitions

A laboratory-based PSG (Alice 4: Respironics Inc., Pittsburgh, USA) was used to diagnose OSA. PSG records were staged manually according to standard criteria by the same skilled technician. Respiratory events were scored according to the American Academic Sleep Medicine (AASM) criteria [18]. The apnea hypopnea index (AHI) was defined as the number of events of apnea and hypopnea per hour during sleep. The parameters of mean oxygen saturation (mean SaO_2_), minimum SaO_2_, the percentage of time spent at an SaO_2_ of <90 % (CT90%), oxygen desaturation index (ODI), and arousal index were also included in the data analysis. Patients with AHI of <5 events/h before LYGB surgery were excluded from the follow-up study. Based on the second PSG test, patients with postoperative AHI of <5 events/h were considered the “cured” group, and those with postoperative AHI of <20 events/h combined with >50 % reduction from baseline were considered the “improved” group.

### Statistical Analysis

Continuous variables are presented as means (standard deviation), except for skewed variables, which are presented as medians (interquartile range). Categorical variables are expressed as percentages. Differences between baseline and postoperative characteristics of the participants were examined using a paired Student’s *t* test, the Wilcoxon signed rank test, the Kruskal-Wallis test, or the *χ* ^2^ test, as appropriate. Correlations between the various variables and PSG parameters were analyzed using Spearman’s correlation test. Parameters that might influence the effect of LYGB surgery on OSA were evaluated by linear regression analysis, with skewed data transformed into normality. We considered *p* values <0.05 to indicate statistical significance for a two-sided test. All statistical analyses were performed using the SPSS software (ver. 13.0.0 for Windows; SPSS Inc., Chicago, IL, USA).

## Results

**Fig. 1:**
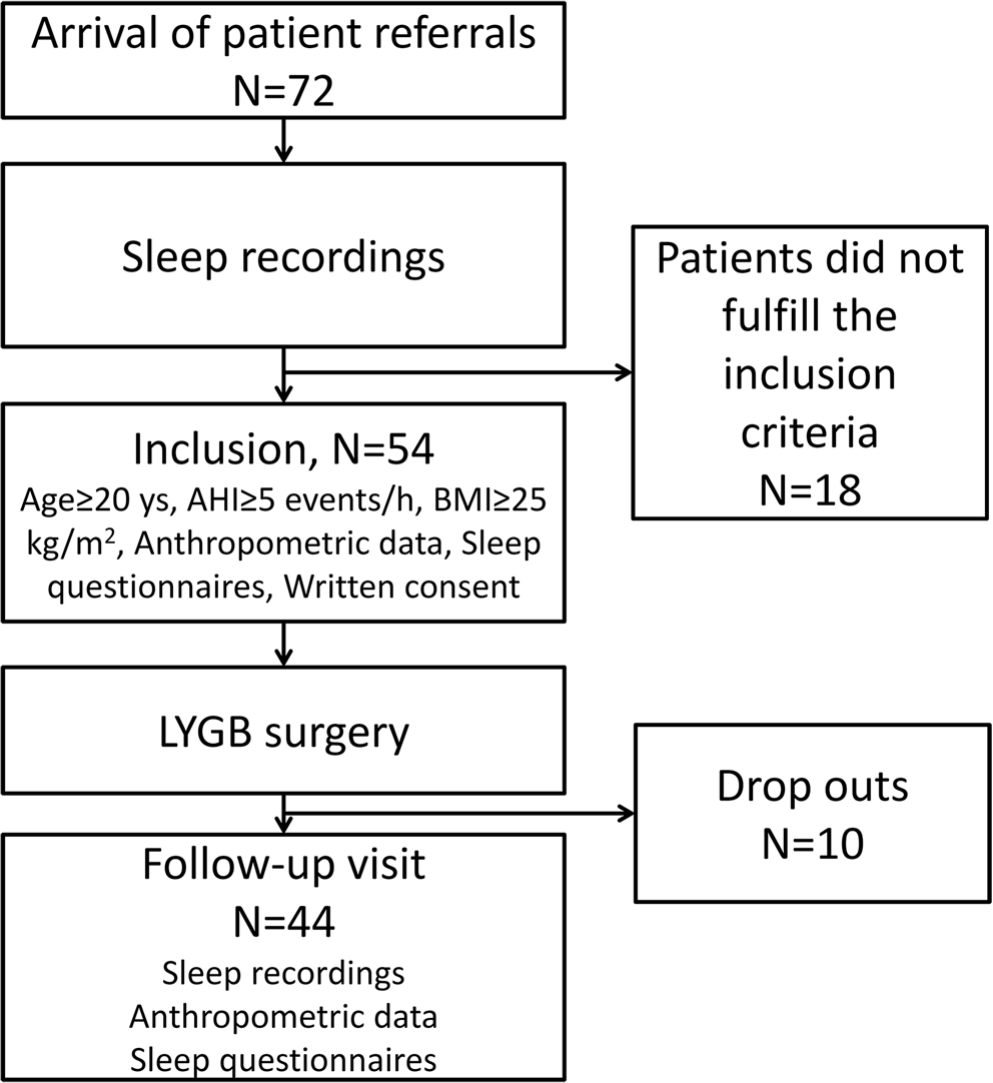
Flow diagram of recruitment of participants. *AHI* apnea hypopnea index, *BMI* body mass index, *LYGB* laparoscopic Roux-en-Y gastric bypass

In total, 72 Chinese patients with obesity and T2DM were recruited for the study. After the first PSG test, 18 subjects were excluded because of AHI <5. The remaining 54 patients with T2DM and OSA were included in the follow-up study. All participants were asked to undergo a second PSG test at least 6 months after surgery. However, ten patients were excluded because of lost to follow-up, or refusing to take the second PSG test (Fig. 1). However, the anthropometric data and sleep recording data from their first visit were similar (*p* > 0.05) to those of the remaining 44 patients. There were 26 (59.1 %) females and 18 (40.9 %) males in the study, aged from 24 to 66 years. The time between the two visits ranged from 6.0 to 24.7 months. Patient demographics are shown in Table 1. The LYGB surgeries were performed by the same team. No adverse event attributable to the LYGB surgery was recorded.

**Table 1:** Overview of the study information

Characteristic	Number (%)	Mean ± SD	Range
Total number of patient	44		
Male	18 (40.9)		
Female	26 (59.1)		
Cured group	28 (63.6)		
Improved group	35 (79.5)		
Height (m)		1.67 ± 0.08	1.53–1.84
Preoperative weight (kg)		83.3 ± 12.7	67.0–131.0
Age (years)		48 ± 11	24–66
Length of follow-up (months)		9.7 ± 5.5	6.0–24.7

### Anthropometric Characteristic Data and Blood Parameters

During the follow-up, the mean change in weight was −18.4 kg (95 % confidence interval [CI], −20.4 to −16.4), and the mean change in BMI was −6.7 kg/m^2^ (95 % CI, −7.4 to −5.9). The prevalence of severe obesity (BMI ≥ 30 kg/m^2^) in the study was 52.3 % at baseline and 2.3 % after the operation. The other measurements of variables at baseline and at the second visit are shown in Table 2. The changes in all of these parameters were significant (*p* < 0.001).

**Table 2:** Comparison of the data associated with anthropometric characteristics, blood measurements, and polysomnography variables

Characteristic	Preoperative (SD)	Postoperative (SD)	Mean difference (95 % CI)	*p* Value^a^
Weight (kg)	86.3 (12.7)	67.9 (10.7)	−18.4 (−20.4 to −16.4)	<0.001
BMI (kg/m^2^)	31.1 (3.4)	24.4 (2.6)	−6.7 (−7.4 to −5.9)	<0.001
NC (cm)	39.6 (2.9)	35.1 (3.4)	−4.5 (−5.3 to −3.7)	<0.001
WC (cm)	105.0 (9.6)	87.7 (9.0)	−17.4 (−19.8 to −15.0)	<0.001
HC (cm)	108.1 (8.6)	95.4 (6.8)	−12.6 (−14.5 to −10.8)	<0.001
W/H ratio	0.97 (0.05)	0.92 (0.07)	−0.05 (−0.07 to −0.03)	<0.001
AHI (/h)	22.4 (17.8)	7.1 (9.4)	−15.4 (−20.3 to −10.4)	<0.001
Apnea index (/h)	10.1 (13.4)	4.4 (8.0)	−5.7 (−9.7 to −1.8)	0.001
Hypopnea index (/h)	12.3 (9.7)	2.7 (3.2)	−9.7 (−12.6 to −6.7)	<0.001
Mean SaO_2_ (%)	93.4 (2.9)	95.5 (1.7)	4.3 (−1.7 to 10.4)	<0.001
Minimum SaO_2_ (%)	77.1 (11.9)	86.7 (6.7)	10.8 (5.7 to 15.8)	<0.001
CT90% (%)	8.0 (12.7)	1.4 (3.2)	−6.7 (−10.4 to −2.9)	<0.001
ODI (events/h)	25.4 (18.6)	6.4 (9.0)	−17.8 (−22.9 to −12.7)	<0.001
Arousal index (/h)	19.8 (13.5)	18.0 (13.4)	−1.4 (−6.0 to 3.2)	0.163
ESS	6.8 (4.7)	3.0 (2.7)	−3.8 (−5.1 to −2.6)	<0.001
Glucose (mmol/L)	8.2 (2.5)	5.7 (1.2)	−2.5 (−3.2 to −1.9)	<0.001
Insulin (uU/mL)	20.6 (17.2)	7.3 (4.9)	−13.3 (−17.8 to −8.8)	<0.001
IR index	1.75 (0.71)	0.46 (0.59)	−1.30 (−1.47 to −1.13)	<0.001

^a^Paired Student’s *t* test for equivalence between groups or Wilcoxon signed rank test for skewed data between groups

### Effect of LYGB on OSA

The mean AHI value was 22.4 events/h (95 % CI, 17.0 to 27.9) at baseline and 7.1 events/h (95 % CI, 4.2 to 9.9) at the second visit. The mean change in AHI was −15.4 events/h (95 % CI, −10.4 to −20.3). The preoperative PSG test showed that 22 patients had mild OSA (5 ≤ AHI ≤ 15 events/h), 9 had moderate OSA (15 < AHI ≤ 30 events/h), and 13 had severe OSA (AHI > 30 events/h). During the follow-up period, 28 (63.6 %) patients were cured of OSA and 35 (79.5 %) patients were improved significantly. The proportions of the mild, moderate, and severe OSA cases who were cured compared to baseline were 77.3, 55.6, and 46.2 %, respectively. The corresponding improved ratios were 77.3, 77.8, and 84.6 %, respectively. The nocturnal oxygen parameters, i.e., mean SaO_2_, minimum SaO_2_, CT90%, and ODI, were also changed significantly (*p* ≤ 0.001). However, the arousal index did not show a meaningful change (*p* = 0.163). Meanwhile, the reported ratio of witnessed apnea and the ESS score also declined significantly (*p* < 0.001).

**Fig. 2:**
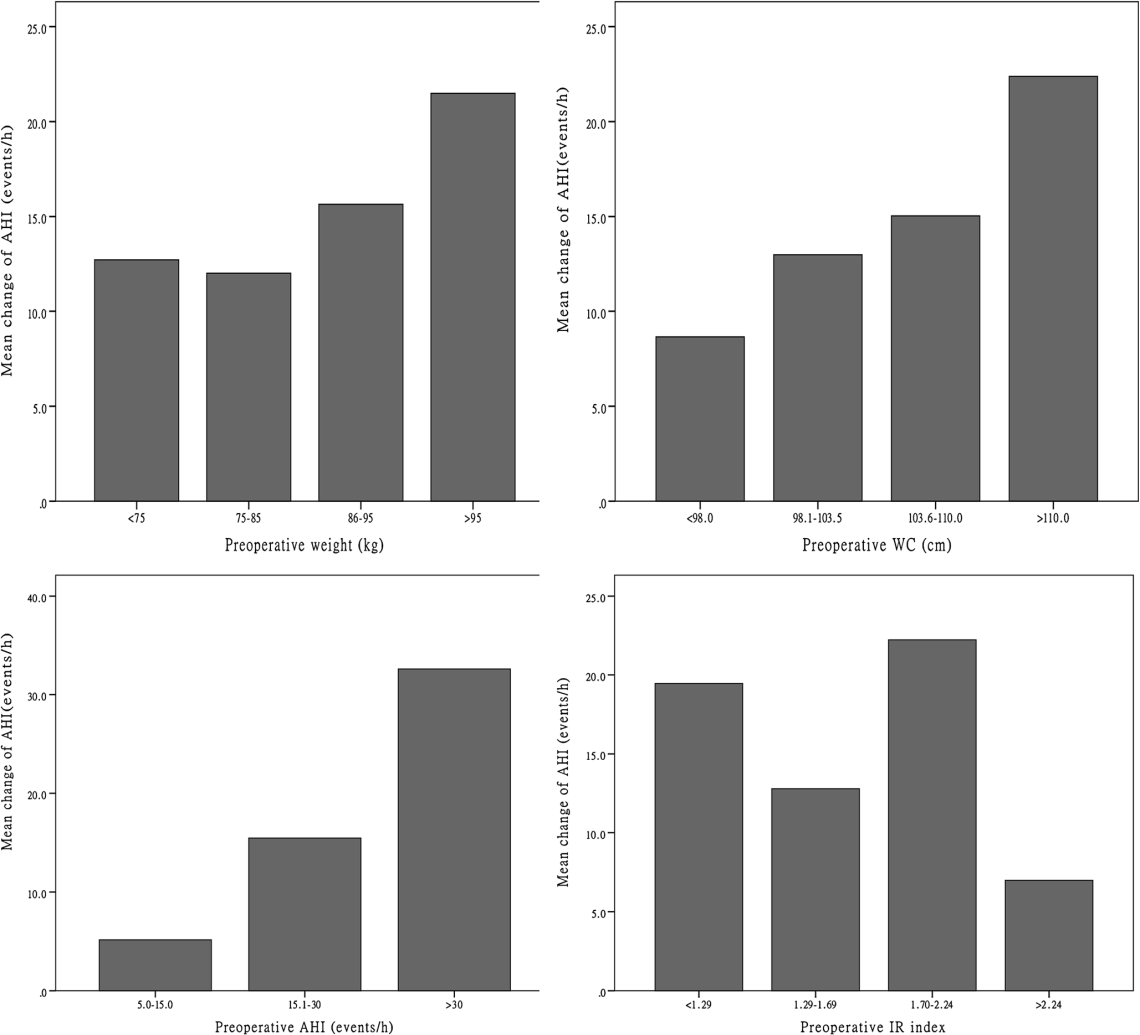
Trends in the change in AHI according to preoperative weight, *WC*, *AHI*, and *IR index. AHI* apnea hypopnea index, *WC* waist circumference, *IR index* insulin resistance index

By Spearman’s rank correlation analysis, the change in AHI was found to be correlated significantly with preoperative weight (*r* = 0.298, *p* < 0.05), preoperative AHI (*r* = 0.729, *p* < 0.001), preoperative WC (*r* = 0.307, *p* < 0.05), and preoperative insulin resistance (IR) index (*r* = −0.301, *p* < 0.05; Fig. 2). In addition to these four parameters, we included gender, age, preoperative BMI, change in BMI, weight reduction, and duration of follow-up to identify other factors associated with the change in AHI, the cured ratio, and the improved ratio. As shown in Table 3, the mean AHI value was significantly reduced in the severe OSA group (*p* < 0.001). The cured ratio showed a downward trend according to increasing AHI, although this was not statistically significant (*p* = 0.15). Moreover, no difference was found between the other groups in the change in AHI, the cured ratio, or the improved ratio.

**Table 3:** Comparison of the changes of AHI between different groups

Group^a^	Number	Change of AHI (95 % CI)	Number of cured	Number of improved
Female	26	−13.1 (−18.7 to −7.4)	16 (61.5 %)	20 (76.9 %)
Male	18	−18.7 (−28.0 to 9.4)	12 (66.7 %)	15 (83.3 %)
Age <40 years	9	−13.3 (−22.3 to −4.4)	8 (88.9 %)	8 (88.9 %)
Age ≥40 years	35	−15.9 (−21.8 to −10.0)	20 (57.1 %)	27 (77.1 %)
PRE-BMI <30.0 kg/m^2^	22	−12.5 (−19.2 to −5.9)	15 (68.2 %)	17 (77.3 %)
PRE-BMI ≥30.0 kg/m^2^	22	−18.2 (−25.8 to −10.6)	13 (59.1 %)	18 (81.8 %)
Mild OSA at baseline	22	−5.15 (−7.0 to −3.3)^b^	17 (77.3 %)	17 (77.3 %)
Moderate OSA at baseline	9	−15.5 (−20.1 to −10.8)	5 (55.6 %)	7 (77.8 %)
Severe OSA at baseline	13	−32.6 (−44.2 to −21.0)	6 (46.2 %)	11 (84.6 %)
PRE-IR index <1	6	−14.1 (−28.1 to −0.1)	4 (66.7 %)	5 (83.3 %)
PRE-IR index ≥1	38	−15.6 (−21.1 to −10.1)	24 (63.2 %)	30 (78.9 %)
Follow-up <1 year	31	−16.4 (−23.2 to −9.7)	20 (64.5 %)	25 (80.6 %)
Follow-up ≥1 year	13	−12.8 (−18.4 to −7.2)	8 (61.5 %)	10 (76.9 %)

^a^The patients were divided into mild OSA group (5 ≤ AHI ≤ 15 events/h), moderate OSA group (15 < AHI ≤ 30 events/h), and severe OSA group (AHI > 30 events/h), based on the preoperative AHI. And, the participants were divided into nonsevere obesity group (BMI < 30 kg/m^2^) and severe obesity group (BMI ≥ 30 kg/m^2^), based on the preoperative BMI. The dividing line was set 40 years for age, 1 for IR index, and 1 year for the follow-up visit. The other parameters were grouped according to the median value

^b^The mean change of AHI was significant among mild OSA group, moderate OSA group, and severe OSA group, *p* value <0.001 with Kruskal-Wallis test

*AHI* apnea hypopnea index, *PRE* preoperative, *BMI* body mass index, *OSA* obstructive sleep apnea, *WC* waist circumference, *IR* insulin resistance

We compared the characteristics between the cured and uncured groups and the improved and unimproved groups. As shown in Table 4, the preoperative AHI was lower in the cured group (*p* = 0.012); however, the values of the mean change in AHI were similar between the two groups (*p* = 0.494). The percent change of AHI was significantly different between the cured group and uncured group (*p* < 0.001) (Supplementary Table 1). In contrast, the preoperative AHI values were similar (*p* = 0.749), and the mean changes in AHI were significantly different (*p* < 0.001) between the improved and unimproved groups (Supplementary Table 2), while the percent change of AHI was significantly different (*p* < 0.001) (Supplementary Table 3).

**Table 4:** Comparisons of data between the cured group and the uncured group

Parameters	Cured group (*n* = 28)			Uncured group (*n* = 16)			*p* value^b^	*p* value^c^
	PRE (SD)	POST (SD)	Mean difference (95 % CI)	PRE (SD)	POST (SD)	Mean difference (95 % CI)		
Total AHI	17.6 (14.9)	2.0 (1.4)^a^	−15.6 (−21.4 to −9.9)	30.8 (19.8)	15.9 (11.0)^a^	−14.9 (−25.0 to −4.9)	0.012	0.494
Age	45.6 (11.8)	46.4 (11.9)	0.8 (0.6 to 1.0)	52.1 (8.6)	52.8 (8.6)	0.8 (0.6 to 1.0)	0.063	0.922
Weight	86.1 (14.1)	67.4 (11.4)^a^	18.8 (−21.6 to −16.0)	86.6 (10.1)	68.9 (9.6)^a^	−17.7 (−20.6 to −14.9)	0.912	0.608
BMI	30.9 (3.5)	24.1 (2.5)^a^	−6.8 (−7.8 to −5.8)	31.4 (3.4)	24.9 (2.8)^a^	−6.5 (−7.5 to −5.4)	0.656	0.671
NC	39.1 (2.8)	35.0 (3.3)^a^	−4.1 (−5.2 to −3.1)	40.5 (2.9)	35.4 (3.7)^a^	−5.1 (−6.4 to −3.8)	0.187	0.239
WC	103.4 (9.4)	86.6 (9.1)^a^	−16.9 (−20.1 to −13.6)	107.9 (9.7)	89.6 (8.9)^a^	−18.3 (−22.2 to −14.3)	0.150	0.572
HC	107.0 (8.2)	95.0 (6.1)^a^	−12.0 (−14.4 to −9.5)	109.9 (9.3)	96.1 (8.1)^a^	−13.8 (−17.0 to −10.7)	0.297	0.350
Glucose	7.9 (2.3)	5.6 (1.2)^a^	−2.3 (−3.1 to −1.5)	8.9 (2.6)	6.0 (1.1)^a^	−2.9 (−4.2 to −1.6)	0.242	0.388
Insulin	19.7 (14.6)	7.2 (5.0)^a^	−12.4 (−17.4 to −7.5)	22.3 (21.4)	7.5 (4.8)^a^	−14.8 (−24.4 to −5.2)	0.981	0.884
IR	1.7 (0.7)	0.4 (0.6)^a^	−1.3 (−1.5 to −1.1)	1.8 (0.8)	0.5 (0.6)^a^	−1.3 (−1.7 to −1.0)	0.522	0.799

^a^Comparisons between the preoperative and postoperative parameters in the cured group or uncured group

^b^Comparisons of the preoperative parameters between the cured group and uncured group

^c^Comparisons between the mean differences of the parameters of the cured group and uncured group

*PRE* preoperative, *POST* postoperative, *AHI* apnea hypopnea index, *AI* apnea index, *HI* hypopnea index, *BMI* body mass index, *NC* neck circumference, *WC* waist circumference, *HC* hip circumference

**Fig. 3:**
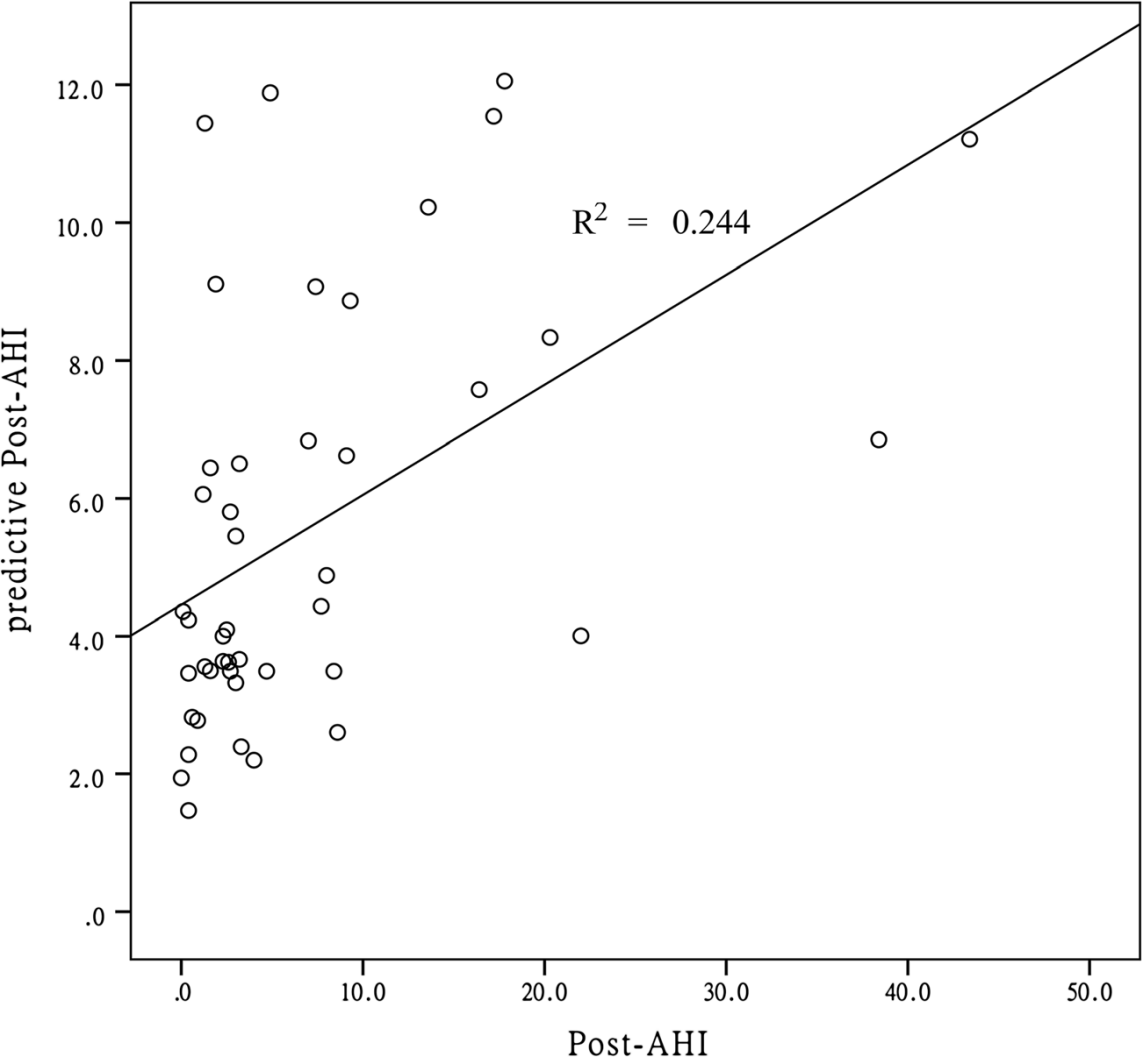
Actual and predicted postoperative AHI values. *AHI* apnea hypopnea index, *Post-AHI* postoperative AHI

When transformed to normality, the postoperative AHI value was correlated significantly with age (*r* = 0.039, *p* = 0.039) and preoperative AHI (*r* = 0.445, *p* = 0.002). By linear regression analysis, we generated a model to predict the postoperative AHI: log_10_(postoperative AHI) = 0.626 × log_10_(preoperative AHI) +0.010 × age −0.581. As shown in Fig. 3, the predictive postoperative AHI was roughly consistent with the trend in the actual postoperative AHI (*r* ^2^ = 0.244, *p* = 0.001); no significant difference was found between the actual value and the predictive value of the postoperative AHI (*p* = 0.870).

## Discussion

In this follow-up study of 44 OSA patients with obesity and T2DM, the total cured ratio and improved ratio of OSA after LYGB surgery were 63.6 and 79.5 %. The cured ratios after surgery in those with mild, moderate, and severe OSA at baseline were 77.3, 55.6, and 46.2 %, respectively. The corresponding improved ratios were 77.3, 77.8, and 84.6 %, respectively. These results indicate that LYGB surgery may be an effective treatment for OSA patients with obesity and T2DM.

Obesity is a known risk factor for the development of OSA [19]. Many studies have been performed to evaluate the effect of bariatric surgery in the treatment of OSA. However, although benefits of bariatric surgery in OSA have been reported, there is no recommendation for this procedure to be included in the clinical guidelines for the treatment of OSA, because studies in sufficiently diverse populations have not been conducted to date, and there is not yet enough evidence to recommend bariatric surgery for treating patients with both OSA and T2DM [20]. Thus, in our study, we focused on the effects of LYGB surgery in a Chinese obesity population with both OSA and T2DM; the outcomes supported the therapeutic efficacy of bariatric surgery in such patients, with 63.6 % of the OSA patients showing resolution and 79.5 % showing significant improvement, similar to a previous report [21]. Moreover, obese patients with severe OSA got more AHI reduction from weight loss than those with only mild or moderate disease (Table 3), consistent with Johansson et al. [22] and Foster et al. [23]. Additionally, the generated prediction model for postoperative AHI might provide a reference method for both surgeons and patients. The improved ratio showed a rising trend according to increasing AHI; however, the cured ratio showed a downward trend according to increasing AHI, probably because of a higher baseline value of AHI. The preoperative AHI value was lower in the cured group, while the mean change in AHI was similar between the two groups. This might mean that patients with less serious OSA have more chance to be cured. And, the similar change of AHI between the cured group and uncured group might be a comprehensive result, because higher AHI reduction happens with the increasing preoperative AHI, but the proportion of more serious OSA at baseline was lower in the cured group. In contrast, the preoperative AHI values were similar while the mean changes in AHI differed significantly between the improved and unimproved groups. Therefore, there might exist some factors, independent of the effect of LYGB surgery, which influence the severity of OSA. However, these contents were not taken into account in the present study.

Weight loss is accompanied by improvements in characteristics related not only to obesity but also to OSA, suggesting that weight loss may be a cornerstone of the treatment of both conditions [24]. However, a relationship between weight reduction and OSA remission has not been demonstrated previously. In our study, the change in AHI after bariatric surgery was correlated only with preoperative weight, AHI, WC, and IR index; no relationship was found between the change in AHI and the change in weight or BMI. In a randomized controlled study, John et al. reported that bariatric surgery did not result in a significantly greater reduction in AHI than conventional weight loss therapy, although the former did result in more weight loss [25]. They suggested that the state rather than the extent of weight loss was important in generating an altered neurohumoral and metabolic-inflammatory milieu, influencing defects in neuromuscular responses to mechanical loads [26, 27]. Furthermore, the modified gastrointestinal anatomy and physiology may influence the effect of bariatric surgery on OSA, although no evidence has been reported to date. Thus, the relationship between weight loss and OSA needs further investigation.

Several limitations of this study should be recognized. First, the duration of follow-up in our study was insufficient. Although a sustained effect of LYGB surgery on weight reduction and OSA improvement has been reported, more recent studies reported weight regain at 5 years after the operation in 50 % of patients [28], with therapeutic failure in 7–20 % at 8 years postoperatively [29]. Thus, modification of OSA through reduction in weight should be followed with a long-term plan. Second, we used data from our observational study, which might have been biased because it relied on patients volunteering for the second polysomnography test, although the preoperative data of the withdrawn group were not significantly different from those of the follow-up group. Third, our study could not provide information about the interaction between bariatric surgery and OSA, due to its simple design. Thus, the underlying mechanisms should be assessed in a future study.

## Conclusions

Our findings indicate LYGB could result in stable and adequate weight loss for obesity patients with both OSA and T2DM, and also reinforce LYGB as an effective therapeutic intervention in the management of OSA in Chinese population. Additionally, the preoperative AHI and age might be important factors that influence the effort of LYGB. However, the underlying mechanisms require further study.

## Electronic supplementary material

Below is the link to the electronic supplementary material.

ESM 1 (PDF 170 kb)

ESM 2 (PDF 115 kb)

ESM 3 (PDF 106 kb)

## References

[1] Young T, Peppard PE, Gottlieb DJ (2002). Epidemiology of obstructive sleep apnea: a population health perspective. Am J Respir Crit Care Med.

[2] Kim J, In K, You S, Kang K, Shim J, Lee S (2004). Prevalence of sleep-disordered breathing in middle-aged Korean men and women. Am J Respir Crit Care Med.

[3] Hiestand DM, Britz P, Goldman M, Phillips B (2006). Prevalence of symptoms and risk of sleep apnea in the US population: results from the national sleep foundation sleep in America 2005 poll. Chest.

[4] Morgenthaler TI, Kapen S, Lee-Chiong T, Alessi C, Boehlecke B, Brown T (2006). Practice parameters for the medical therapy of obstructive sleep apnea. Sleep.

[5] Sarrell EM, Chomsky O, Shechter D (2013). Treatment compliance with continuous positive airway pressure device among adults with obstructive sleep apnea (OSA): how many adhere to treatment?. Harefuah.

[6] Sin DD, Mayers I, Man GC, Pawluk L (2002). Long-term compliance rates to continuous positive airway pressure in obstructive sleep apnea: a population-based study. Chest.

[7] Ferguson KA, Cartwright R, Rogers R, Schmidt-Nowara W (2006). Oral appliances for snoring and obstructive sleep apnea: a review. Sleep.

[8] Barnes M, McEvoy RD, Banks S, Tarquinio N, Murray CG, Vowles N (2004). Efficacy of positive airway pressure and oral appliance in mild to moderate obstructive sleep apnea. Am J Respir Crit Care Med.

[9] Hoekema A, Stegenga B, De Bont LG (2004). Efficacy and co-morbidity of oral appliances in the treatment of obstructive sleep apnea-hypopnea: a systematic review. Crit Rev Oral Biol Med.

[10] Elshaug AG, Moss JR, Southcott AM, Hiller JE (2007). An analysis of the evidence-practice continuum: is surgery for obstructive sleep apnoea contraindicated?. J Eval Clin Pract.

[11] Koutsourelakis I, Georgoulopoulos G, Perraki E, Vagiakis E, Roussos C, Zakynthinos SG (2008). Randomised trial of nasal surgery for fixed nasal obstruction in obstructive sleep apnoea. Eur Respir J.

[12] Sundaram S, Bridgman SA, Lim J, Lasserson TJ (2005). Surgery for obstructive sleep apnoea. Cochrane Database Syst Rev.

[13] De Dios JA, Brass SD (2012). New and unconventional treatments for obstructive sleep apnea. Neurotherapeutics.

[14] Buchwald H, Avidor Y, Braunwald E, Jensen MD, Pories W, Fahrbach K (2004). Bariatric surgery: a systematic review and meta-analysis. JAMA.

[15] Krieger AC, Youn H, Modersitzki F, Chiu YL, Gerber LM, Weinshel E (2012). Effects of laparoscopic adjustable gastric banding on sleep and metabolism: a 12-month follow-up study. Int J Gen Med.

[16] Regional Office for the Western Pacific (WPRO) WHO, International Association for the Study of Obesity, International Obesity Task Force. The Asia-Pacific perspective: redefining obesity and its treatment. 2000.

[17] World Health Organization. Definition, diagnosis and classification of diabetes mellitus and its complications: report of a WHO consultation. Part 1: diagnosis and classification of diabetes mellitus. 1999.

[18] Iber C, Ancoli-Israel S, Chesson AL, Quan SF (2007). For the American Academy of Sleep Medicine. The AASM manual for the scoring of sleep and associated events: rules, terminology and technical specifications.

[19] Fritscher LG, Mottin CC, Canani S, Chatkin JM (2007). Obesity and obstructive sleep apnea-hypopnea syndrome: the impact of bariatric surgery. Obes Surg.

[20] Chang CL, Marshall NS, Yee BJ, Grunstein RR. Weight-loss treatment for OSA: medical and surgical options. Eur Respir Mon. 2010:302–20.

[21] Sarkhosh K, Switzer NJ, El-Hadi M, Birch DW, Shi X, Karmali S (2013). The impact of bariatric surgery on obstructive sleep apnea: a systematic review. Obes Surg.

[22] Johansson K, Neovius M, Lagerros YT, Harlid R, Rossner S, Granath F (2009). Effect of a very low energy diet on moderate and severe obstructive sleep apnoea in obese men: a randomised controlled trial. BMJ.

[23] Foster GD, Borradaile KE, Sanders MH, Millman R, Zammit G, Newman AB (2009). A randomized study on the effect of weight loss on obstructive sleep apnea among obese patients with type 2 diabetes: the sleep AHEAD study. Arch Intern Med.

[24] Romero-Corral A, Caples SM, Lopez-Jimenez F, Somers VK (2010). Interactions between obesity and obstructive sleep apnea: implications for treatment. Chest.

[25] Dixon JB, Schachter LM, O’Brien PE, Jones K, Grima M, Lambert G (2012). Surgical vs conventional therapy for weight loss treatment of obstructive sleep apnea: a randomized controlled trial. JAMA.

[26] Patil SP, Schneider H, Schwartz AR, Smith PL (2007). Adult obstructive sleep apnea: pathophysiology and diagnosis. Chest.

[27] Schwartz AR, Patil SP, Laffan AM, Polotsky V, Schneider H, Smith PL (2008). Obesity and obstructive sleep apnea: pathogenic mechanisms and therapeutic approaches. Proc Am Thorac Soc.

[28] Magro DO, Geloneze B, Delfini R, Pareja BC, Callejas F, Pareja JC (2008). Long-term weight regain after gastric bypass: a 5-year prospective study. Obes Surg.

[29] Valezi AC, Mali Junior J, de Menezes MA, de Brito EM, de Souza SA (2010). Weight loss outcome after silastic ring Roux-en-Y gastric bypass: 8 years of follow-up. Obes Surg.

