# Supplementary material for: Effect of Laparoscopic Roux-en-Y Gastric Bypass Surgery on Obstructive Sleep Apnea in a Chinese Population with Obesity and T2DM
Source: Obes Surg. 2014 Nov 14;25(8):1446–53. doi: 10.1007/s11695-014-1510-9 (PMC4498416; doi:10.1007/s11695-014-1510-9)
Supplement: Supplementary file 1 — (PDF 170 kb) [file 11695_2014_1510_MOESM1_ESM.pdf]

Supplementary table 1. Comparisons of the percent change of the parameters between the cured group and the uncured group

parameters	Cured group (n=28)		Uncured group (n=16)		p-value*
	Mean difference (95% CI)	Percent change (95% CI)	Mean difference (95% CI)	Percent change (95% CI)	
Total AHI	-15.6 (-21.4 to -9.9)	82.15 (74.83 to 89.48)	-14.9 (-25.0 to -4.9)	36.32 (15.30 to 57.35)	<0.001
Age	0.8 (0.7 to 1.0)	1.42 (0 to 2.84)	0.8 (0.6 to 1.0)	2.02 (0.20 to 3.85)	0.253
Weight	-18.8 (-21.6 to -16.0)	21.38 (18.47 to 21.29)	-17.7 (-20.6 to -14.9)	20.47 (17.52 to 23.41)	0.603
BMI	-6.8 (-7.8 to -5.8)	21.38 (18.47 to 24.28)	-6.5 (-7.5 to -5.4)	20.47 (17.52 to 23.41)	0.604
NC	-4.1 (-5.2 to -3.1)	10.06 (7.55 to 12.57)	-5.1 (-6.4 to -3.8)	12.61 (9.55 to 15.67)	0.268
WC	-16.9 (-20.1 to -13.6)	15.85 (12.95 to 18.75)	-18.3 (-22.2 to -14.3)	16.82 (13.54 to 20.09)	0.745
HC	-12.0 (-14.4 to -9.5)	10.87 (8.71 to 13.02)	-13.8 (-17.0 to -10.7)	12.45 (9.86 to 15.04)	0.372
Glucose	-2.3 (-3.1 to -1.5)	24.98 (16.96 to 33.00)	-2.9 (-4.2 to -1.6)	28.60 (18.45 to 38.74)	0.614
Insulin	-12.4 (-17.4 to -7.5)	55.70 (46.08 to 65.31)	-14.8 (-24.4 to -5.2)	55.44 (42.16 to 68.72)	0.904
IR	-1.3 (-1.5 to -1.1)	80.42 (65.04 to 95.79)	-1.3 (-1.7 to -1.0)	75.31 (60.36 to 90.26)	0.635

*p-value for the comparison of percent change between the cured group and the uncured group.

AHI=apnoea-hypopnoea index; BMI=body mass index; NC=neck circumference; WC=waist circumference; HC=hip circumference; IR=insulin resistance index
