# Supplementary material for: Effect of Laparoscopic Roux-en-Y Gastric Bypass Surgery on Obstructive Sleep Apnea in a Chinese Population with Obesity and T2DM
Source: Obes Surg. 2014 Nov 14;25(8):1446–53. doi: 10.1007/s11695-014-1510-9 (PMC4498416; doi:10.1007/s11695-014-1510-9)
Supplement: Supplementary file 2 — (PDF 115 kb) [file 11695_2014_1510_MOESM2_ESM.pdf]

Supplementary table 2. Comparisons of data between the improved group and the unimproved group

parameters	Improved group (n=35)			Unimproved group (n=9)			p-value&	p-value*
	PRE (SD)	POST (SD)	Mean difference (95% CI)	PRE (SD)	POST (SD)	Mean difference (95% CI)		
Total AHI	22.7 (18.9)	3.9 (4.5)#	-18.7 (-24.4 to -13.1)	21.5 (13.6)	19.2 (13.5)	-2.3 (-6.2 to 1.6)	0.749	<0.001
Age	47.2 (11.5)	48.0 (11.6)	0.8 (0.7 to 1.0)	51.0 (9.2)	51.7 (9.3)	0.7 (0.5 to 0.9)	0.362	0.549
Weight	86.8 (13.3)	68.0 (11.2)#	-18.8 (-21.2 to -16.4)	84.3 (10.4)	67.5 (8.9)#	-16.8 (-20.2 to -13.4)	0.607	0.428
BMI	31.2 (3.5)	24.4 (2.8)#	-6.8 (-7.7 to -5.9)	30.7 (3.2)	24.5 (2.2)#	-6.2 (-7.6 to -4.8)	0.695	0.391
NC	39.7 (3.2)	35.5 (3.7)#	-4.3 (-5.3 to -3.3)	39.1 (1.4)	33.9 (1.7)#	-5.2 (-5.9 to -4.4)	0.548	0.100
WC	105.2 (10.4)	87.8 (9.7)#	-17.4 (-20.4 to -14.4)	104.4 (6.6)	87.2 (6.5)#	-17.2 (-20.4 to -14.0)	0.837	0.884
HC	108.2 (8.6)	95.6 (7.0)#	-12.6 (-14.8 to -10.3)	107.7 (9.2)	94.8 (6.4)#	-12.9 (-16.5 to -9.3)	0.881	0.953
Glucose	8.1 (2.4)	5.7 (1.2)#	-2.4 (-3.1 to -1.7)	8.9 (2.8)	6.0 (1.2)#	-2.9 (-5.0 to -0.7)	0.389	0.850
Insulin	18.6 (13.7)	7.1 (4.6)#	-11.5 (-15.7 to -7.3)	28.5 (26.7)	8.2 (6.1)#	-20.2 (-37.1 to -3.3)	0.127	0.239
IR	1.7 (0.6)	0.4 (0.6)#	-1.3 (-1.4 to -1.1)	2.0 (0.9)	0.6 (0.7)#	-1.4 (-2.0 to -1.0)	0.173	0.274

Comparisons between the preoperative and postoperative parameres in the improved group or unimproved group.

& Comparisons of the preoperative parameters between the improved group and unimproved group.

* Comparisons between the mean differences of the paremeters of the improved group and unimproved group.

PRE=preoperative; POST=postoperative; AHI=apnoea-hypopnoea index; BMI=body mass index; NC=neck circumference; WC=waist circumference; HC=hip circumference
