# Supplementary material for: Effect of Laparoscopic Roux-en-Y Gastric Bypass Surgery on Obstructive Sleep Apnea in a Chinese Population with Obesity and T2DM
Source: Obes Surg. 2014 Nov 14;25(8):1446–53. doi: 10.1007/s11695-014-1510-9 (PMC4498416; doi:10.1007/s11695-014-1510-9)
Supplement: Supplementary file 3 — (PDF 106 kb) [file 11695_2014_1510_MOESM3_ESM.pdf]

Supplementary table 3. Comparisons of the percent change of the parameters between the improved group and the unimproved group

parameters	Improved group (n=35)		Unimproved group (n=9)		p-value*
	Mean difference (95% CI)	Percent change (95% CI)	Mean difference (95% CI)	Percent change (95% CI)	
Total AHI	-18.7 (-24.4 to -13.1)	80.38 (74.48 to 86.29)	-2.3 (-6.2 to 1.6)	8.93 (-14.8 to 32.66)	<0.001
Age	0.8 (0.7 to 1.0)	1.85 (1.47 to 2.24)	0.7 (0.5 to 0.9)	1.47 (1.08 to 1.85)	0.140
Weight	-18.8 (-21.2 to -16.4)	21.49 (19.03 to 23.95)	-16.8 (-20.2 to -13.4)	19.92 (16.72 to 23.12)	0.534
BMI	-6.8 (-7.7 to -5.9)	21.49 (19.02 to 23.95)	-6.2 (-7.6 to -4.8)	19.92 (16.72 to 23.12)	0.535
NC	-4.3 (-5.3 to -3.3)	10.70 (8.35 to 13.04)	-5.2 (-5.9 to -4.4)	13.23 (11.26 to 15.20)	0.284
WC	-17.4 (-20.4 to -14.4)	16.34 (13.76 to 18.91)	-17.2 (-20.4 to -14.0)	16.48 (13.62 to 19.34)	0.956
HC	-12.6 (-14.8 to -10.3)	11.44 (9.52 to 13.35)	-12.9 (-16.5 to -9.3)	11.81 (9.06 to 14.56)	0.850
glucose	-2.4 (-3.1 to -1.7)	26.20 (19.45 to 32.94)	-2.9 (-5.0 to -0.7)	28.17 (12.66 to 43.69)	0.790
Insulin	-11.5 (-15.7 to -7.3)	54.52 (46.06 to 62.97)	-20.2 (-37.1 to -3.3)	61.94 (44.64 to 79.24)	0.981
IR index	-1.3 (-1.4 to -1.1)	79.10 (66.48 to 91.72)	-1.4 (-2.0 to -1.0)	76.81 (56.72 to 96.91)	0.864

*p-value for the comparison of percent change between the improved group and the unimproved group.

AHI=apnoea-hypopnoea index; BMI=body mass index; NC=neck circumference; WC=waist circumference; HC=hip circumference; IR=insulin resistance
